# Dissecting the Molecular Signature of Spinal Cord Regeneration in the Axolotl Model

**DOI:** 10.7759/cureus.7014

**Published:** 2020-02-16

**Authors:** Turan Demircan

**Affiliations:** 1 Genetics, Muğla Sıtkı Koçman University, Muğla, TUR

**Keywords:** axolotl, regeneration, spinal cord injury, spinal cord regeneration, transcriptomics, omics

## Abstract

Thousands of people are affected by central nervous system (CNS) dysfunctions each year, with stroke and spinal cord injury (SCI) being the most frequent causes. Although there is some evidence of partial CNS self-repair (via migration of neural stem cells to the injury zone and adult neurogenesis), due to restricted regeneration capacity in mammals, acute or chronic spinal cord injuries cannot be repaired completely. Therefore, to expand the availability of treatment options for SCI, research on highly regenerative animals has become essential. Among vertebrates, axolotl, a salamander species, has been emerging as a powerful model to explore the molecular mechanisms of regeneration due to its exceptional regenerative capacity. In this study, gene expression modulation for regenerative-capable neotenic axolotl during spinal cord regeneration has been investigated. Next-generation sequencing was applied for the collected regeneration samples at zero and seven days post-amputation (dpa). The data obtained from the analyzed samples revealed 363 genes differentially expressed, mostly downregulated, between zero dpa and seven dpa. The extracellular matrix, cell-cell adhesion, and immune system-related processes and pathways were enriched by gene ontology and the Kyoto Encyclopedia of Genes and Genomes (KEGG) pathway enrichment analyses. Based on these data, we conclude that the downregulation of immune system-related biological processes is crucial for spinal cord regeneration.

## Introduction

Spinal cord injury (SCI) is one of the more common acute central nervous system (CNS) injuries seen throughout the world. Currently, limited treatment options are available for spinal cord repair and regeneration [1]. Lack of or insufficient axonal regrowth and the formation of glial scars are the main characteristics of wound healing following an SCI in humans [2]. The very quick physiological response to the acute injury following the initial damage is the restrictive point to the development of effective therapies for SCI, and therefore, medical interventions to prevent the effect of secondary injury is the typical approach to SCI treatment [3]. Functional consolidation of the CNS after an injury in some species such as zebrafish and salamanders has attracted the attention of researchers to decipher the regenerative capacity code in these animals [4]. Furthermore, the lack of a successful clinical therapy option for SCI cases promotes the usage of animal models to understand the molecular mechanisms involved in the course of spinal cord regeneration.

Among vertebrates, the amphibian axolotl (*Ambystoma mexicanum*), also known as Mexican salamander, possesses an exceptional regeneration capacity compared to other species at the adult stage [5]. Axolotls can regenerate their internal organs, brain, spinal cord, limb, and tail [6]. This remarkable regenerative potential has been attributed to axolotl life-long neoteny, characterized by the exhibition of embryonic characteristics at the adult stage. As other noteworthy experimental features, scar-free wound healing and astonishingly low cancer incidence are associated with the regenerative capability of the axolotl [7-8]. Regeneration in axolotl is characterized by three main steps: wound healing, neural-epithelial interactions, and reprogramming of positional information [9]. When a part of an axolotl is damaged or amputated, the release of pro-regenerative signals to the damaged zone attracts epithelial cells to close the wound and form the wound epithelium. This step is followed by the infiltration of immune system elements (e.g., macrophages) to the damaged area [10]. Removal of pathogens and cell debris by immune cells provides the formation of the apical epithelial cap as a consequence of keratinocytes; this is linked to innervation. As a complementary biological process, differentiated cells undergo dedifferentiation via the activity of nerve-dependent gene expression, resulting in the secretion of regulatory signals from keratinocytes. As a result, differentiated cells gain division capacity and stem/progenitor cells start to accumulate in the wound area to form a regenerative specific tissue called blastemal. This crucial step is followed by redifferentiation to provide functional restoration [11].

In this study, we explored the molecular mechanisms in spinal cord regeneration by utilizing the axolotl model. Introducing a spinal cord injury in neotenic axolotls was followed by collecting samples at zero (control) and seven days post-amputation (dpa), the blastema/regeneration time point. Gene expression analysis of these samples pinpointed the biological processes that have a suppressive or stimulating effect on spinal cord regeneration. This work constitutes one of the very first efforts to chart the axolotl gene expression profile during spinal cord regeneration.

## Materials and methods

**Figure 1:**
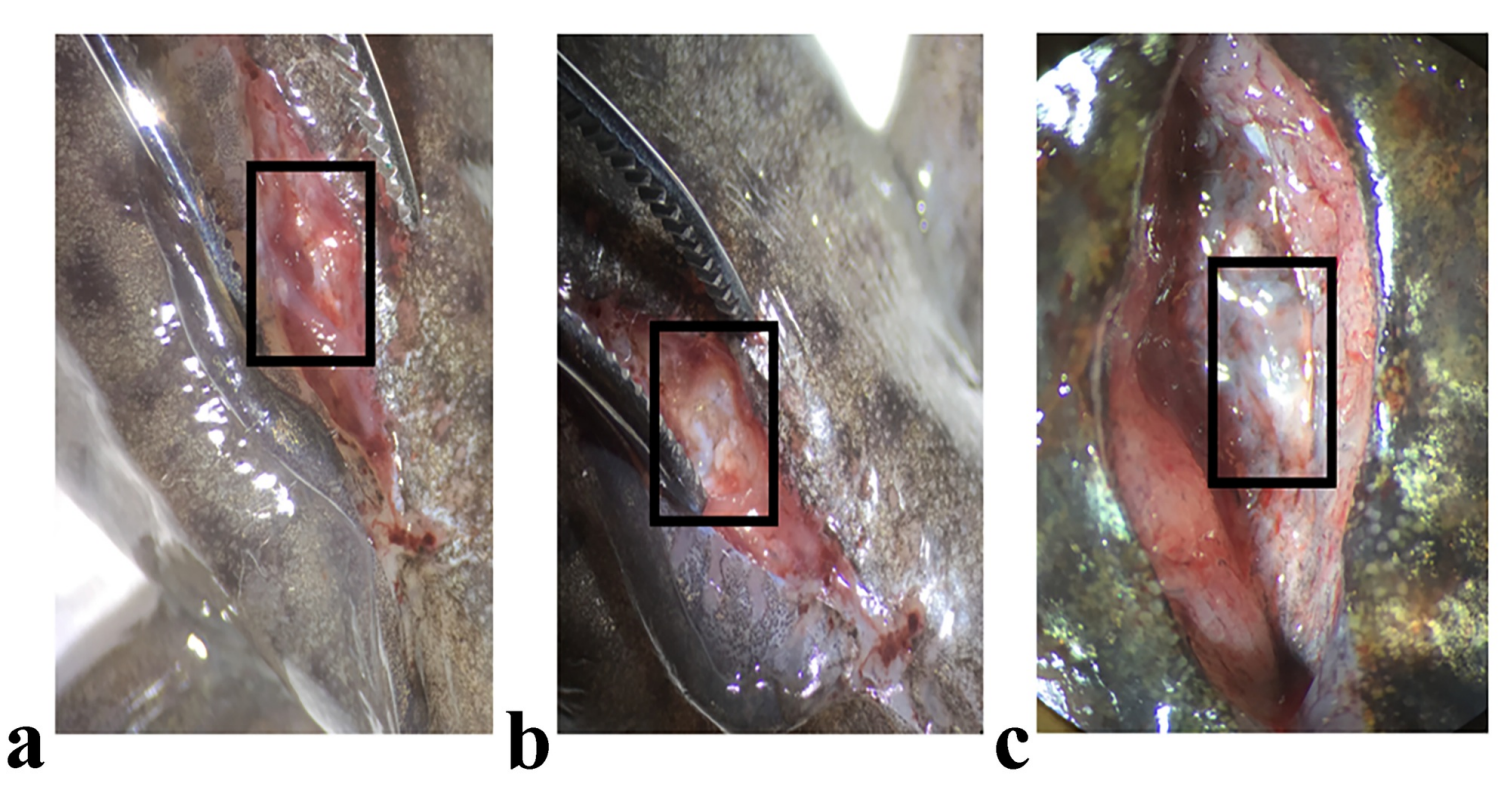
Axolotl spinal cord injury model Spinal cord injury was introduced in axolotls; a) just after the laminectomy, b) shortly after the spinal cord removal procedure, c) four weeks after operation when spinal cord regeneration took place

Axolotls (6-8 months old, 15 siblings) used in the experiments were bred in the Istanbul Medipol University Animal Care Facility. The followed procedures were approved by the Istanbul Medipol University Animal Experiments Local Ethical Committee (authorization number 38828770-E.2499). Animals were maintained at 18°C in 20% Holtfreter’s solution, kept in chambers as one animal per aquarium, and fed once a day using staple food (JBL Novo; JBL GmbH & Co. KG, Neuhofen, Germany) throughout the experimental period as described elsewhere [12]. Axolotls were anesthetized in 0.1% benzocaine (E1501 SIGMA; Merck KGaA, Darmstadt, Germany) for all applied experimental procedures. Animals were randomly grouped to have three biological replicates (R1, R2, and R3) by having five animals in each replicate. All animals were operated above the position of hindlimb as described previously [13]. Following the laminectomy, the exposed spinal cord in between two consecutive vertebrae were extracted to form the zero dpa samples (Figure 1). After the operation, animals were put back to their chambers and blastema samples were collected to form the seven dpa samples.

RNAs were extracted from the collected samples using Trizol reagent (ThermoFisher Scientific, Waltham, MA; catalog no: 1559601) according to the manufacturer’s protocol. The RNA quantities were determined by Qubit assay according to the manufacturer’s protocol (ThermoFisher Scientific, catalog no: Q33140). For each replicate, the RNA concentration was measured at a concentration greater than 200 ng/µl. The quality of isolated RNAs was checked on 1% agarose gel. After quantity and quality controls, a TrueSeq Stranded messenger RNA (mRNA) kit (Illumina, San Diego, CA; catalog no: 20020594) was used for mRNA library preparation by following the manufacturer’s protocol. The Illumina NextSeq 500 platform was employed for RNA sequencing by using a NextSeq 500/550 High Output Kit v2.5 150 cycles (Illumina, catalog no: 20024907).

Demultiplexing and barcode removal for the paired-end sequencing dataset obtained from samples was performed using the Illumina BaseSpace platform. The resulting barcode-free raw sequencing data were retrieved and preprocessed using Illumina FASTQC software, followed by Cutadapt software [14-15]. While FASTQC was used for quality control, Cutadapt was used for quality filtering. In the quality filtering step, only high-quality (minimum average Phred quality score of 30) reads passed; reads below this threshold were discarded. The remaining sequences were used as input for the Trinity software for de novo assembly [16]. Annotations of assembled RNA sequences were found via the Basic Local Alignment Search Tool (BLAST) searches using the National Center for Biotechnology Information (NCBI) transcripts database. To find the differentially expressed genes between zero and seven dpa, the DESeq2 method was applied [17]. To perform enrichment analyses, most similar mouse RNA orthologs were identified by running a BLAST search against the NCBI mouse transcript database. For downstream analyses, redundant genes and genes with no matching mouse orthologue were filtered out.

Gene ontologies and Kyoto Encyclopedia of Genes and Genomes (KEGG) pathway enrichment analyses of the list of differentially expressed (DE) genes between seven dpa and zero dpa neotenic samples were queried using the clusterProfiler R/Bioconductor package [18,19]. Visualization of the gene ontologies and pathways enriched by those DE genes was performed using a bar plot, dot plot, and a network plot, all of which were implemented using the same R package (version 3.6.1). The parameters of the enrichment analysis included setting P and Q value cutoffs to 0.05, org.Mm.eg.db (mouse) as the organism database, and Benjamini & Hochberg (BH) as the adjusted p-value method.

## Results

A total of 363 genes were found to be differentially expressed after running DESeq2 analyses. To inspect those genes with the most significant fold change (FC), genes having an FC ranging between 2 and -2 were selected, leaving a total of six upregulated and 346 downregulated genes remaining after filtering. Afterward, genes that had no matching mouse orthologue were filtered out, yielding a total of 148 genes. Genes were then queried to get their corresponding EntrezID that was then used in the downstream enrichment analysis, yielding a total of 140 matched genes. Some of the accession numbers redundantly corresponded to the same gene ID, and so they were accounted for by taking the mean of the corresponding FC, resulting in a total of 129 genes. The final list of genes used for enrichment consisted of two upregulated and 127 downregulated genes (Table 1).

**Table 1:** List of differentially expressed genes at seven days post-amputation compared to zero dpa: days post-amputation

Condition	Gene symbol
Upregulated genes at seven dpa	Cpe, Nrcam
Downregulated genes at seven dpa	Actb,Adamts4, Adgrg2, Anxa1, Apcs, Apoh, Arg1, Arl11, Arrdc2, Atf3, Atp12a, Birc5, Btn1a1, Camp, Cbfb, Cblif, Ccdc71l, Ccn1, Cd63, Cd68, Cebpb, Ch25h, Chia1, Cirbp, Coq10b, Ctf2, Cxcr2, Dusp5, Dusp7, Eef1d, Eif4a1, Emp1, Etnppl, Fbp1, Fhdc1, Fmc1, Fn1, Fosb, Fosl1, Fstl1, Fth1, Gadd45g, Gm10997, Gm11175, Gm13889, Gm7808, Hif3a, Hsp90b1, Ier3, Ifitm6, Igfbp1, Il10ra, Il11, Il1b, Il6, Junb, Kcng3, Keap1, Klf2, Klf5, Krt75, L1Md-Tf23, Lamc2, Larp6, Lpar6, Lpo, Lrg1, Lrrc58, Lta, Lum, Mas1, Mitd1, Mmp13, Mmp19, Mmp3, Mmp9, Mog, Mrc1, Nfil3, Ngp, Noct, Ociad2, Padi3, Pdpn, Pgls, Plau, Plbd1, Plin2, Ppan, Pprc1, Prg3, Prss27, Psca, Rpl19, Rpl21, Rpl31-ps1, Rpl5, Rplp1-ps1, Rps17, Rundc3a, Runx1, Sds, Selp, Serpinb5, Slc1a5, Snai2, Sorbs3, Sqstm1, Ssr4, Stk17b, Susd6, Tent5a, Tgif1, Tgm2, Tinagl1, Tnf, Tnfaip2, Tnfrsf12a, Traf1, Traf3, Trex2, Upp1, Vcp, Vmp1, Wnt9a, Xbp1, Xirp1

**Figure 2:**
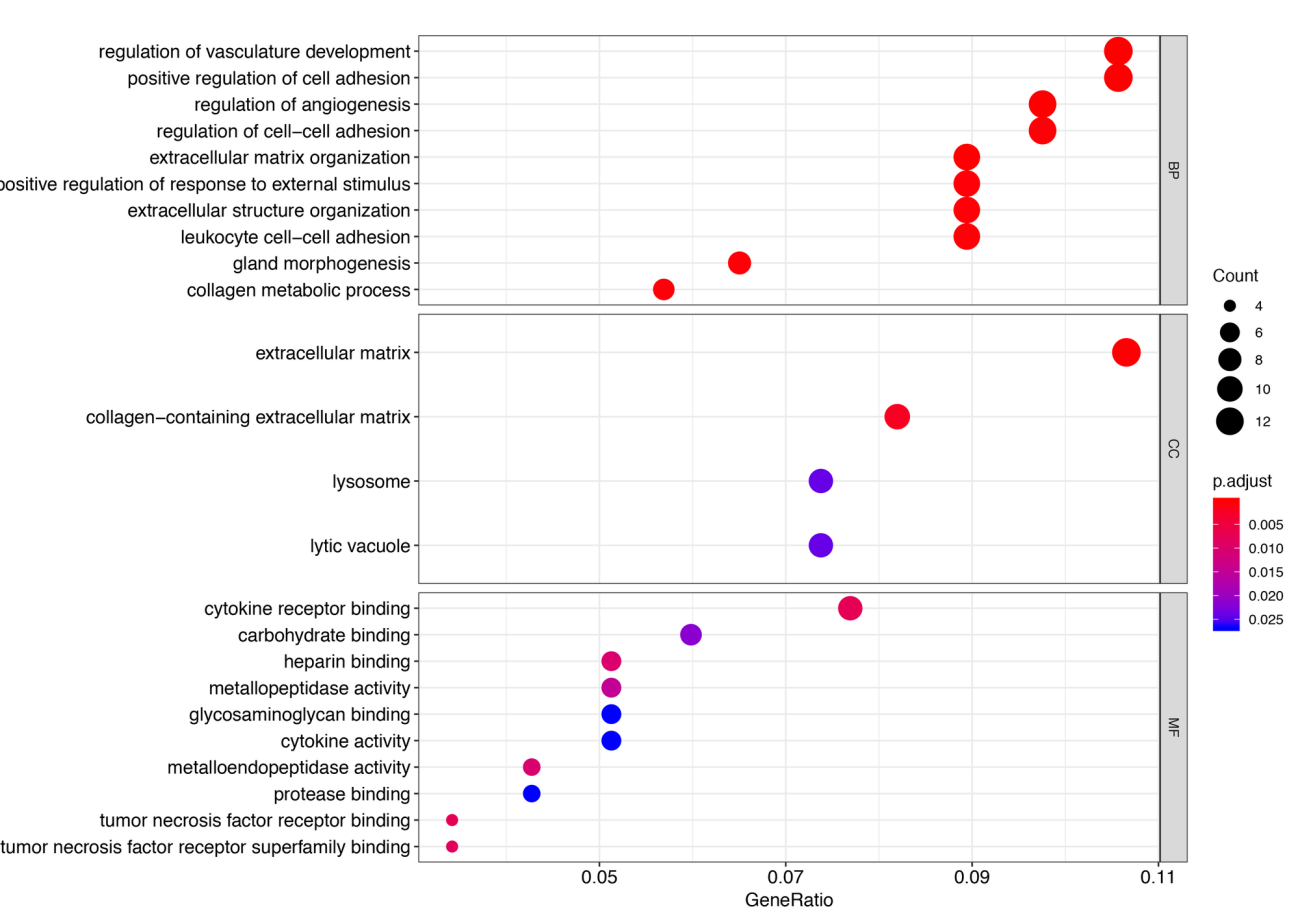
Gene Ontology enrichment analysis The top 10 biological processes (BP) and molecular functions (MF) enriched by the DE genes are depicted by a dot plot, in addition to the only four enriched cellular components (CC), sorted by the most significant. The colored bar represents the adjusted p-value, with the sizes of the dots being proportional to the enriched gene counts

Many of the DE genes in neotenic seven dpa vs. zero dpa samples were enriched in several biological processes; the top 10 of these are visualized in Figure 2. Examples include areas such as regulation of vasculature development, positive regulation of cell adhesion, regulation of angiogenesis, and regulation of cell-cell adhesion. A total of four cellular components were only found enriched by those genes, namely the extracellular matrix (ECM), collagen-containing extracellular matrix, lysosome, and lytic vacuole (Figure 2). Moreover, cytokine receptor binding, carbohydrate-binding, heparin-binding, and metallopeptidase activity were amongst the top 10 molecular functions enriched by the DE genes (Figure 2). Importantly, all of the processes were dominantly enriched by downregulated genes.

**Figure 3:**
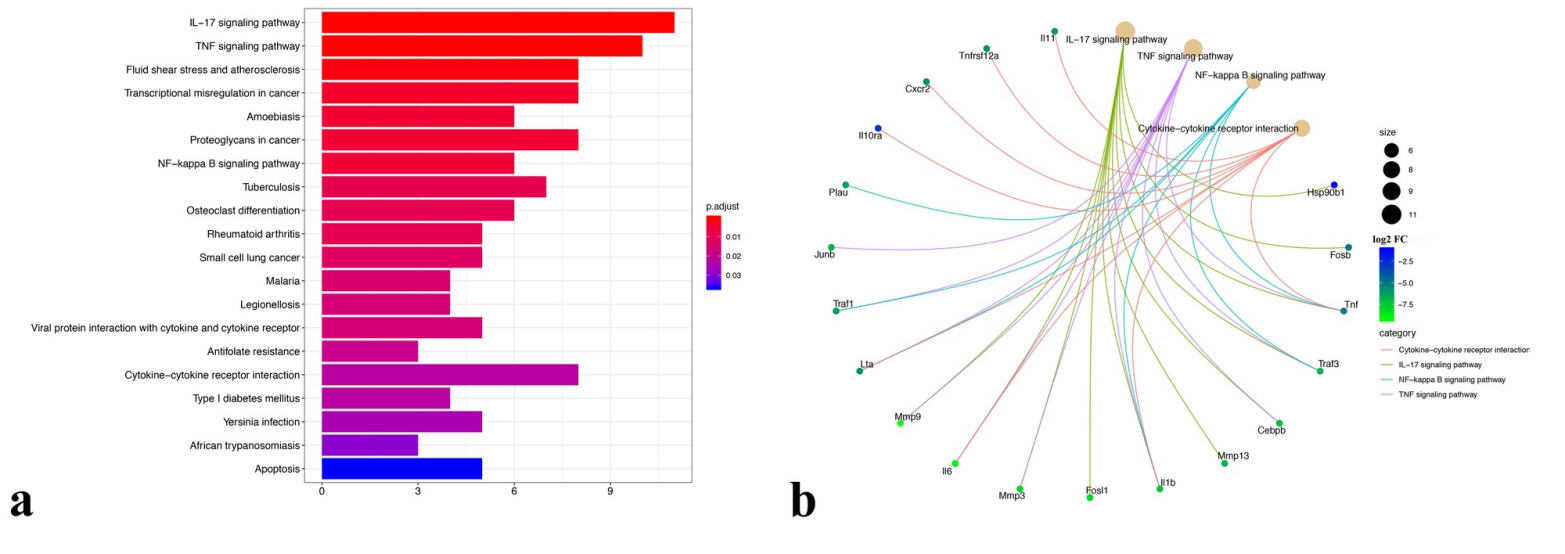
Kyoto Encyclopedia of Genes and Genomes pathway enrichment analysis a) All of the enriched Kyoto Encyclopedia of Genes and Genomes (KEGG) pathways are depicted in a bar plot with a color scale representing the adjusted p-value sorted by the most significant. b) A cnetplot depicting the immunity-related pathways along with the corresponding enriched genes. The edges are colored corresponding to the pathway name. Gene nodes are colored according to their log2 fold change (FC). The size of the pathway nodes is proportional to the gene count

Enrichment of the DE genes in neotenic seven dpa versus zero dpa samples was evident in several pathways, such as fluid shear stress and atherosclerosis, transcriptional misregulation in cancer, amoebiasis, proteoglycans in cancer, as well as 16 other pathways (Figure 3A). Interestingly, out those 20 pathways, four immunity-related pathways were found enriched by a set of the DE genes, namely the IL-17 signaling pathway, tumor necrosis factor (TNF) signaling pathway, NF-kappa B signaling pathway, and cytokine-cytokine receptor interaction (Figure 3B). These were all enriched by some genes unique to them in addition to two common ones (Tnf, Il1b). Similar to Gene Ontology (GO) analysis, pathways were dominantly enriched by downregulated genes.

## Discussion

SCI is still a devastating neurological disorder, and exploring the regulatory factors of spinal cord regeneration is an important scientific question to address. Despite the growing body of regeneration-related research on mammalian model organisms, due to limited regenerative capacity in mammals, the comprehensive profiling and elucidation of key players in this process have remained largely elusive. However, highly regenerative animal models such as axolotl offer an excellent system to study spinal cord regeneration. In this respect, the presented work is a step towards filling this research gap.

Most of the DE genes were found as downregulated at seven dpa as compared to zero dpa. Interestingly these genes were enriched mainly in cell-cell or cell-ECM interaction related biological processes such as positive regulation of cell adhesion, regulation of cell−cell adhesion, leukocyte cell−cell adhesion, and extracellular matrix organization. As shown in previous studies, for successful regeneration, the dedifferentiation of specialized cells, cell division, and cell migration processes provided by the reorganization of cell-cell and cell-ECM adhesion capacity are required to facilitate the formation of blastema tissue [11,20]. Thus, enrichment of downregulated genes at seven dpa in these biological processes aligned well with previous studies demonstrating the impact of ECM remodeling in the regeneration of the axolotl spinal cord and limb [21-22]. This notion is further supported by enrichment of extracellular matrix and collagen-containing extracellular matrix terms in cellular components by downregulated genes at seven dpa, indicating degradation and/or reorganization of ECM.

Another intriguing observation of the analyzed data was the enrichment of immune system-related KEGG pathways such as the IL-17 signaling pathway, TNF signaling pathway, NF-kappa B signaling pathway, and cytokine-cytokine receptor interactions by the downregulated genes at seven dpa, which is underlying the decreased activity of immune system at blastema stage during spinal cord regeneration. In previous studies, it has been postulated that the enhanced activity of the immune system may interfere with a regenerative capacity [23]. Therefore, from a translational medicine perspective, in combination with current approaches, suppression of excessive inflammatory reactions in spinal cord injury may hold promise for a better healing process as suggested by others [24].

The limitations of this study included an analysis based on a single time point of regeneration. Multiple time points in the course of regeneration would have provided more insights into the molecular regulation of spinal cord regeneration. The current study is further limited by a lack of functional studies that are required to arrive at mechanistic explanations for spinal cord regeneration.

## Conclusions

This is a pioneering study on axolotl spinal cord regeneration that aims to explore the molecular and cellular mechanisms of spinal cord regeneration. In this study, we observed that regulation of cell-cell adhesion, ECM organization, and immune activity might be important for successful regeneration. Further follow-up validation and characterization research, particularly on the impact of the immune system during regeneration, could provide new insights into understanding spinal cord regeneration and possibly advance the development of regenerative therapies for injuries and disabilities related to SCI in mammals.
